# Chest Pain With Significantly Elevated Troponins: Be Wary of False Positives

**DOI:** 10.7759/cureus.77018

**Published:** 2025-01-06

**Authors:** Taarunya T Narayanan, Tamara Naneishvili, William Moody, John Townend, Peter Ludman

**Affiliations:** 1 Department of Cardiology, Queen Elizabeth Hospital, Birmingham, GBR

**Keywords:** assay interferance, atypical chest pain, covid-19 vaccine, false-positive troponins, macrotroponin

## Abstract

High-sensitivity cardiac troponins are considered a gold standard for diagnosing acute myocardial infarction and myocardial injury. However, the occurrence of false positives needs to be kept in mind.

We describe the clinical challenges in diagnosing a 45-year-old woman who repeatedly presented to the emergency department with atypical chest pain and extremely elevated high sensitivity troponin I (HsTnI), despite normal imaging including cardiac MRIs and invasive coronary angiograms, on multiple occasions.

This report emphasizes the importance of carefully interpreting elevated troponin levels, especially when clinical findings and further investigations do not support a cardiac origin for troponin (Tn) elevation.

## Introduction

Myocardial infarction remains one of the foremost causes of mortality worldwide. It affects 3.8% of individuals under 60 years and an alarming 9.5% of those over 60, with prevalence rates varying across different regions [1]. In the United Kingdom alone, myocardial infarction accounts for approximately 100,000 hospital admissions annually. Thanks to advancements in diagnostic and treatment techniques, around 1.4 million individuals have survived this potentially fatal event [2].

Innovations in diagnostic algorithms, particularly the development of high-sensitivity troponins (HsTn), have revolutionized the approach for early rule-in or rule-out myocardial infarction in the emergency department, thereby improving survivability. These assays can detect troponins at remarkably low concentrations, offering precise measurements that are crucial for predicting the severity of myocardial injury and providing valuable prognostic information [3,4]. However, despite their high sensitivity, these assays can yield positive results in healthy individuals.

The following case report explores the clinical challenges and diagnostic complexities encountered in a patient presenting with persistently elevated high-sensitivity troponin I (HsTnI) levels and atypical chest pain without evidence of myocardial injury.

## Case presentation

A 45-year-old Asian woman presented to the emergency department in late 2021 with sudden onset, localized left-sided chest pain that was not related to exertion. Her pain was sharp and stabbing in nature, with no positional variation. She had no symptoms of palpitations, diaphoresis, shortness of breath, or fevers and no history of a recent infection. She had received a Pfizer COVID booster vaccine 24 hours before her presentation. She had a past diagnosis of antiphospholipid antibody (APLA) syndrome, for which she was on aspirin during her pregnancy in 2005. A repeat APLA screen in 2014 was indeterminate, with low levels of IgG and IgM, and negative lupus anticoagulant and Beta-2 glycoprotein 1 antibody (B2GPlb) screen. She also had a history of Arnold Chiari malformation, cervical syringomyelia, and scalp folliculitis. She smoked seven cigarettes a day but never drank alcohol. She had no family history of premature coronary heart disease. Her brother had coronary artery disease (CAD) and stenting at the age of 55, and her father had a coronary artery bypass graft (CABG) when he was 78 years old.

Her physical examination and vital signs were normal. Her initial 12-lead ECG showed a normal sinus rhythm with a rate of 70 beats per minute, with no changes suggesting ischemia. High-sensitivity troponin I assays were done sequentially, and both the values were >6000ng/L (cut off for women <16ng/L). Her D-dimer, C-reactive protein (CRP), and ESR were negative (Table 1).

**Table 1:** Relevant biochemical investigations. HsTnI: High sensitivity troponin I; HsTnT: High sensitivity troponin T; CK: Creatinine kinase; NT-ProBNP: N-terminal pro b-type natriuretic peptide; CRP: C-reactive protein; ESR: Erythrocyte sedimentation rate; eGFR: Estimated glomerular filtration rate. All reference ranges are defined based on local trust protocols, considering the sex, age, and ethnicity of the specific case, wherever applicable.

	HsTnI (ref. range: 0-16 ng/L)	HsTnT (0-14 ng/L)	CK (< 200 IU/L)	NT-ProBNP (<400pg/ml)	CRP (<5mg/L)	ESR (<29 mm/hr)	eGFR (>90ml/min)
11/2019	6	-	-	-	2	-	>90
3/12/21	6526	-	-	-	-	-	>90
3/12/21	6467	-	-	-	-	-	>90
13/7/23	2886	-	-	-	<1	-	>90
13/7/23	3235	-	-	-	-	-	>90
29/7/23	3879	-	-	28	<1	-	>90
9/8/23	2662	-	80	-	<1	2	>90
24/8/23	2764	-	-	-	-	-	>90
28/8/23	3019	-	-	-	<1	-	>90
11/10/23	1575	-	-	-	-	-	>90
19/11/23	2012	-	114	-	<1	-	>90
19/11/23	1987	-	-	17	-	5	>90
28/12/23	1226	147	3	-	-	-	-
17/1/24	2040	-	138	-	-	-	>90
3/3/24	2552	-	-	-	<1	5	>90
3/3/24	2813	-	-	-	-	-	>90

**Figure 1:**
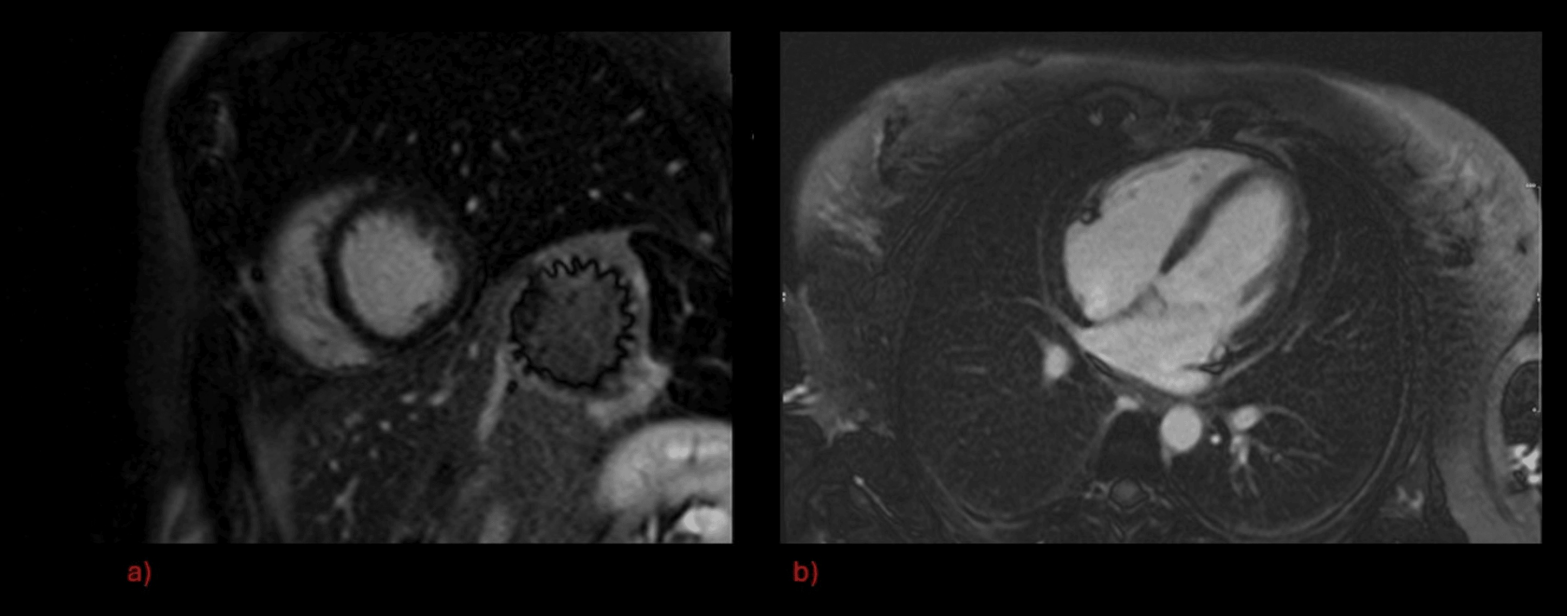
Gadolinium enhanced cardiac MRI during first in-patient episode

A transthoracic echocardiogram showed normal left ventricular function with no regional wall motion abnormalities. At this stage, her differential diagnosis was felt to be either myocarditis or acute coronary syndrome, though her symptoms were atypical. A cardiac MRI did not show any late gadolinium enhancement (Figure 1a, Figure 1b).

**Figure 2:**
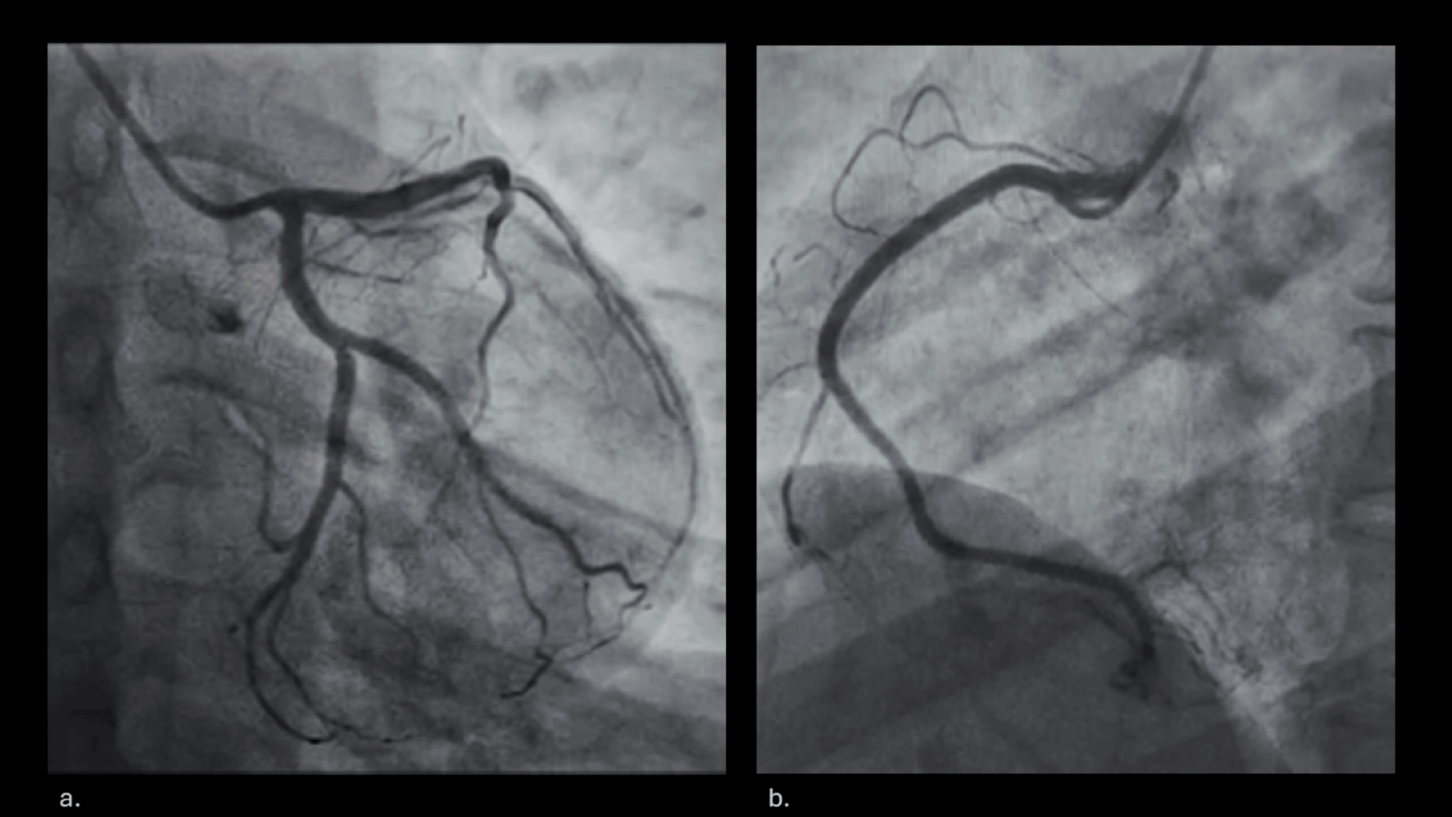
Invasive coronary angiogram.

Invasive coronary angiography did not show any evidence of coronary artery disease (Figure 2a, Figure 2b).

**Figure 3:**
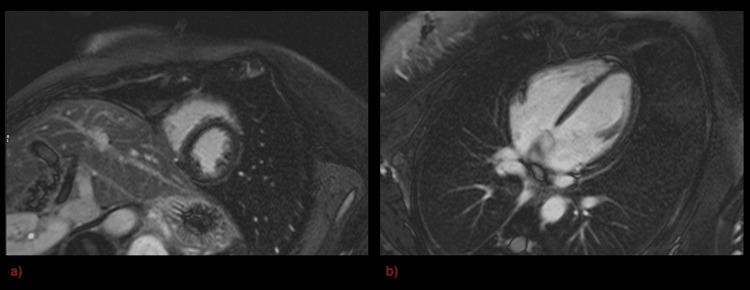
Repeat gadolinium enhanced cardiac MRI

She was discharged, with a repeat cardiac MRI arranged in six weeks. Again, this did not show any abnormalities, including late gadolinium enhancement (Figure 3a, Figure 3b). 

**Figure 4:**
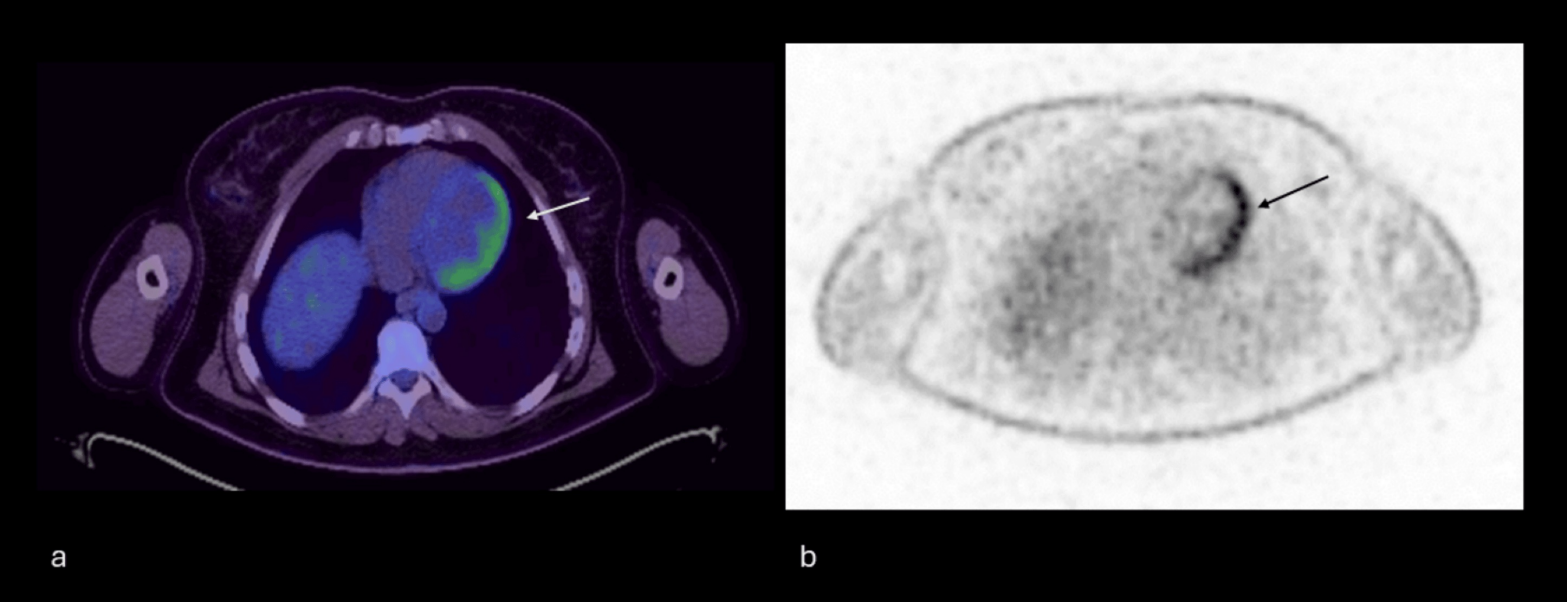
PET-CT images.

Two years later, in 2023, she re-presented to the emergency department with recurrent sharp, central chest pain at rest. There were no pleuritic or pericarditic features. Again, her physical examination and 12-lead ECG remained normal, but her HS troponin I was elevated at >2800 ng/L. Her CRP and ESR were negative (Table 1). Myocarditis or acute coronary syndrome was once again considered to be the most likely diagnosis. However, a CT coronary angiogram showed no evidence of flow-limiting coronary atheroma. A subsequent repeat cardiac MRI remained normal, with no late gadolinium enhancement. PET-CT was performed (Figure 4a, Figure 4b).

This showed some focal heterogeneous increase in tracer uptake involving the left ventricular apex as well as the lateral wall. The patient adhered to a high-fat, zero-carbohydrate diet for the day preceding the FDG-PET with a fast of 12 hours and a pre-scan blood glucose of 4.6 mmol/L. Extensive serology and immunology tests were undertaken for suspected myocarditis. Her serology showed a positive IgG to Borrelia (P58 antigen); however, all other IgG and IgM antibodies were negative. Tests for IL 1 antibodies related to myocarditis post-COVID vaccine returned an equivocal result [5]. 

**Figure 5:**
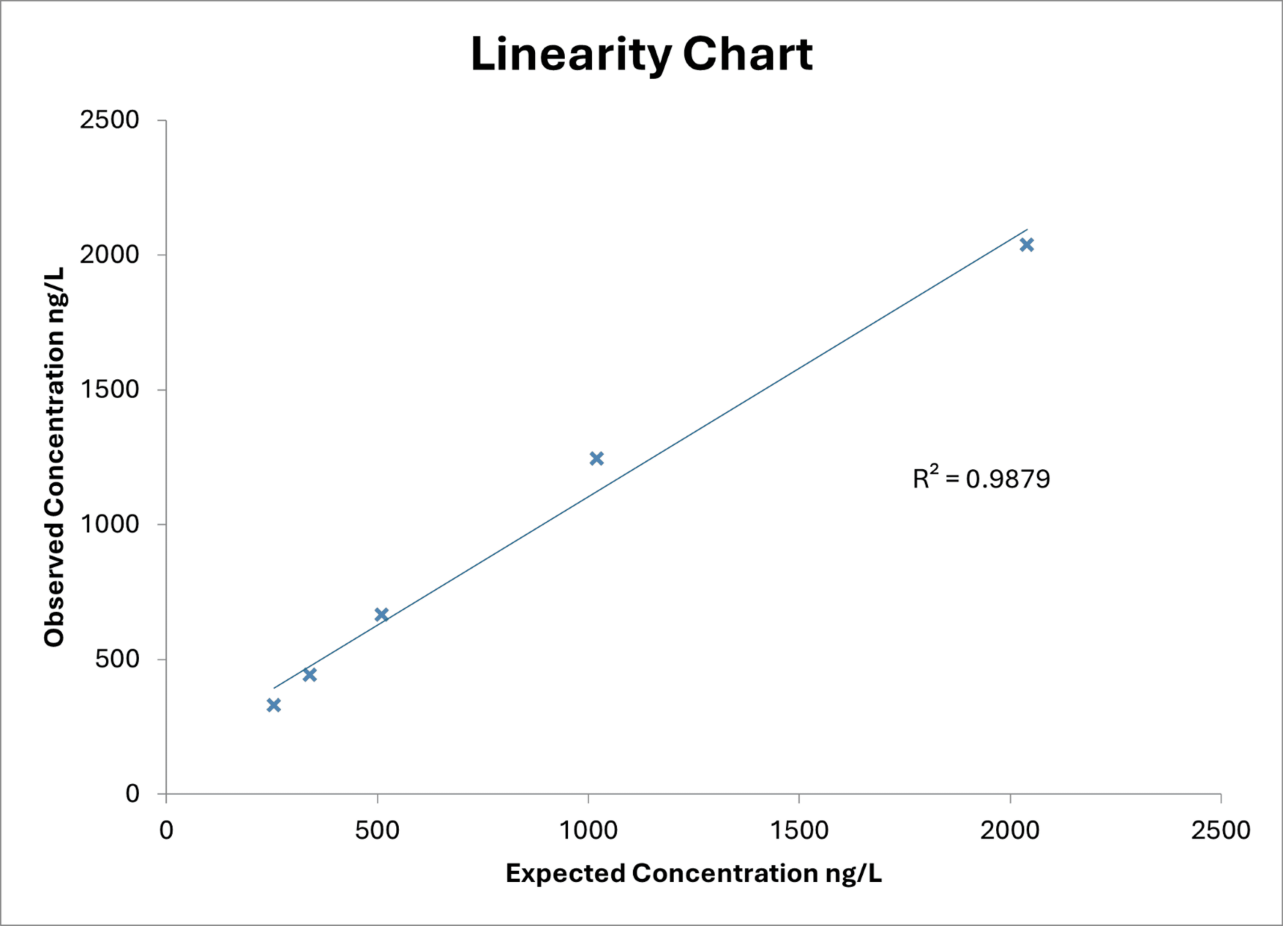
Results of the linearity experiment.

In view of the biochemical and PET imaging evidence of potential active myocarditis, she was started on a trial of high-dose methylprednisolone. This was discontinued due to intolerance. At this stage, a further opinion was sought. The isolated rise in her HsTnI, with a normal troponin T and only marginally high creatinine kinase, raised the suspicion that her very high HsTnI levels were due to a false positive test result and not secondary to myocardial injury or infarction. Investigations, including the linearity experiment and polyethylene glycol precipitation (PEG) test, were done to identify the cause of the suspected false positive results. The linearity experiment showed that the troponin concentration diluted linearly in the sample, thereby excluding interference due to heterophilic antibodies, matrix effects, or prozone errors (Figure 5).

A PEG test revealed that her troponin recovery was <0.4%, thus proving the presence of macro troponin complexes. On further review, it was felt that her PET-CT appearances were false positives. Her presenting symptoms were, therefore, unrelated to cardiac pathology.

## Discussion

We describe the case of a patient whose very elevated levels of HsTnI resulted in numerous investigations into the possibility of myocarditis or acute coronary syndrome. HsTnI measurement is so embedded in the investigation and management pathways of patients presenting to emergency departments, that, as in this case, unexpected causes for this may be missed.

Cardiac troponins are detected using immunochemical methods such as enzyme-linked immunosorbent assay (ELISA), immunofluorescence assay, radioimmunoassay, and immunochemiluminescence assay. These assays involve an initial immunological phase, where an antibody-antigen reaction to cardiac Tn occurs, followed by an enzymatic reaction, and a detection phase using a spectrophotometer (ELISA), radiometer (RIA), or fluorometer (immunofluorescence assays). Modern assays have been refined to minimize cross-reactivity, with fifth-generation immunoassays now representing the gold standard for determining cardiac TnT. Similar advancements have been made for cardiac TnI [6].

Despite its excellent biochemical sensitivity and its status as the gold standard for identifying and prognosticating myocardial infarction, multiple instances of false-positive troponins have been documented in the literature. Heterophilic antibodies are produced in response to antigens such as blood transfusions, vaccinations, persistent viral infections, and autoantibodies produced in autoimmune conditions such as rheumatoid arthritis (RA), systemic lupus erythematosus (SLE), and polymyositis [7].

The underlying mechanism for immune-mediated false-positive troponin involves in-vivo or in-vitro antibodies. These complexes lead to persistent high troponins due to their slower clearance from the system. The polyethylene glycol (PEG) precipitation test is used to assess the presence of circulating macro troponin. The addition of PEG precipitates these high molecular weight molecules. After centrifugation, the measured and recovery rate of troponin is calculated. A low recovery rate suggests the presence of macro troponins [8]. The PEG test is routinely used to test for macro prolactin, however, it is being adapted to test for macro troponins [9].

There have been case reports of COVID-19 infection and vaccines as a potential cause for macrotroponinemia [10,11]. In the case described above, the patient had normal HsTnI prior to the vaccination, followed by a significant troponin rise within 24 hours of vaccination. Although she did have an underlying autoimmune pathology, this was disproven by further testing for APLA. Therefore, the COVID-19 vaccine could probably be a cause for her persistent macrotroponinemia. Despite persistent elevation in troponins, the all-cause and cardiovascular mortality caused by macrotroponinemia alone is significantly low when compared to patients with a true increase in troponin [12]. It can, however, lead to misdiagnosis and over-investigation as in this case.

## Conclusions

High-sensitivity troponins are central to the investigation of patients presenting with chest pain to emergency departments. They are extremely valuable in aiding early diagnosis and guiding the management of patients with cardiac disease. Nevertheless, it is crucial to be aware of the limitations of this test, and as with any investigation, a HsTnI result should not be considered in isolation. The clinical context and other findings must always be considered. If the test does not seem to fit with these, then other causes of raised HsTnI measurements need to be considered, and it is important to remember the possibility of false positive results caused by macro troponin complexes and heterophile antibodies.
